# One pot sets another boiling: A case of social learning perspective about leader self-serving behaviour and followers self-serving counterproductive work behaviour

**DOI:** 10.1016/j.heliyon.2023.e14611

**Published:** 2023-03-18

**Authors:** Uzma Sarwar, Sidrah Al Hassan, Osama Khassawneh, Tamara Mohammad, Rashida Parveen

**Affiliations:** a School of Education, Huanggang Normal University, Huanggang, 438000, PR China; b Department of Business Administration, Faculty of Management Sciences, International Islamic University Islamabad(IIUI), Pakistan; c The Emirates Academy of Hospitality Management, United Arab Emirates; d American University in the Emirates, Dubai, United Arab Emirates

**Keywords:** Leader self-serving behaviour, Self-serving cognitive distortions, Self-serving counterproductive work behaviour, Follower machivellianism, Social learning theory

## Abstract

Self-Serving leadership is a global phenomenon and requires both literary and practical attention to understand how it unfolds and impacts organizations. More specifically the investigation of this underexplored dark side of leadership in Pakistani service sector organizations has its unique significance. So, in this regard, the current study took the initiative to investigate the relationship between a Leader's self-serving behaviour and a follower's self-serving counterproductive work behaviour. Moreover, the underlying mechanism of self-serving cognitive distortions was proposed, with followers' Machivellianism strengthening the indirect relationship between leaders' self-serving behaviour with self-serving counterproductive work behaviour through the self-serving cognitive distortions. The proposed theoretical framework was explained by the Social Learning theory. This study adopted a survey method with the collection of data by utilizing the convenience sampling method, in three-time waves with peer-reported self-serving counterproductive work behaviours. The data was analyzed by utilizing confirmatory factor analysis to establish discriminant and convergent validity. Moreover, the hypotheses testing was done utilizing Hayes Process Macro 4 (Mediation) and 7(Moderated Mediation). The results proved that the self-serving cognitive distortions mediated the relationship between the leader's self-serving behaviour and the follower's self-serving counterproductive work behaviours. Moreover, it was established that the High Mach tendencies strengthed the indirect positive relationship between a leader's self-serving behaviour with self-serving counterproductive work behaviour through the self-serving cognitive distortions. It is important to note that the current study provides a view to the practitioners that formulation of effective policies and systems for identifying and discouraging the tendencies of Leaders' self-serving behaviour and employing people with low Mach Tendencies can avoid the self-serving counterproductive work behaviours harming the overall welfare of the organization.

## 1 Introduction

The dark side of leadership investigating the counterproductive forms of leader behaviour has got lesser empirical attention in the existing literature [1]. This negative side of leadership highlights different leadership styles (like abusive supervision and exploitative leadership) that have positive correlations with self-serving leader behaviour. But, it should be kept in view that this specific leader behaviour is distinct from these aforementioned styles [2]. This behaviour highlights leaders' indulgence in furthering their self-interests to gain benefits for themselves [3]. In other words, this leader's behaviour generally does not address the collective interest and harms both employees and stakeholders in the organizational setting. But despite its long-held existence, it still is an understudied phenomenon [2]. So, this leader behaviour needs scholarly attention[1] as such low-intensity and goal-oriented behaviours lead to employee deviance. In particular, this behaviour of the leader results in terrible outcomes for both the employees and the organization [4]. Hence, the investigation of self-serving leader behaviour is important for the empirical investigation to highlight its negative repercussions for an organization's welfare.

Dark leadership behaviours like self-serving leadership are more prevalent in Asian countries and there is a clear need to study such underexplored areas of research in this region [5]. Although, various organizational scandals like the Famous Enron Scandal [6] indicate that it is a long-held global phenomenon but the investigation of self-serving leadership in Pakistani service sector organizations is worth investigation due to the dearth of empirical evidence from this region [5]. It has been evidenced that dark leadership is more prevalent in cultures that are characterized by high uncertainty avoidance, collectivism, and high power distance [7]. In this regard, it is important to note that Pakistan is a South Asian country that rates relatively high on power distance, masculinity, short-term orientation, and uncertainty avoidance [8]. So such features highlight the fact that in such a culture the leader may be more prone to indulge in material gains accomplishing self-interest with little chance of being held accountable for such actions. The reason for it is that the actions of people in authority figures are seldom held questionable in such cultures [9].

In response to a self-serving leader, the followers are reported to indulge in negative behaviours, like increased organizational deviance, and interpersonal deviance [10], a high desire for retaliation, a stronger urge for supervisor-directed deviance [11], and an upward surge in deviant work behaviours [12]. Moreover, it has been recently advised that specific leadership styles can foster self-serving counterproductive work behaviours (CWB) [13]. Therefore, the first aim of the current study was to propose that self-serving leader behaviour can instigate self-serving CWB. This specific form of CWB is characterized by covert, fraudulent, embezzlement, and self-interest behaviours, which pose harm to both organizations and individuals. More specifically, the self-serving CWB is defined as “Acts of deviance, which are demonstrated in an attempt to further one's self-interest at the cost of counterparts or organization. They are thoughtfully planned and executed in a covert and subtle manner in an attempt to hide one's ulterior motives” [13]. So, in a Pakistani culture characterized by high power distance, in response to a leader's self-serving behaviour, the employees will learn to indulge in such forms of CWB as they will consider their superior's behaviour as fair and acceptable and take them as their role model for emulation. Hence, it can be understood that in organizations operating in a high power distance culture, the superiors are considered as the focus of attention and as a source of social learning [14].

The recent literature on self-serving leader behaviour answers the question of “why” employees indulge in negative work behaviours like organizational deviance and interpersonal deviance by investigating organizational identification as an underlying mechanism [15]. However, another study highlights that in response to the self-centred acts of the leader, their subordinates may learn (see social learning theory Bandura, 1986) to hide the knowledge and this destroys their team's creativity [16]. On the other hand, “how” employees indulge in different behaviours while observing self-serving leaders has been answered in the literature by empirically establishing that the activation of moral disengagement [17]results in subordinates learning [18] to exhibit deviant workplace behaviour [12]. So, in the light of existing literature, the second aim of the current study was about answering “how” observing a self-serving leader results in the adoption of self-serving CWB. Hence, the current study proposed the unique underlying mechanism of self-serving cognitive distortions in the relationship between self-serving leader behaviour and self-serving CWB. It is relevant to mention that these self-serving cognitions operate at the primary and secondary levels. At the primary level, distortions take the form of self-centred attitudes and beliefs, whereas at the secondary level distortions are characterized as rationalizations that neutralize the moral consciousness and avoid the loss of self-image [19]. Self-serving cognitions are biased ways of giving meaning to experiences that lead to wrong and aggressive behaviours[5]. There are four main categories of self-serving cognitive distortions that include Self-Centred, Blaming Others, Minimizing/Mislabeling, and Assuming the Worst. Self-centred distortions are attitudes characterized by neglecting or disrespecting others' opinions and need while focusing on one's own opinions, needs, and rights. Blaming others comprises cognitive schemas characterized as shifting blame for one's behaviour to other people. Mislabeling involves acts that demean others. Assuming the Worst signifies cognitive distortions where the individual attributes antagonistic intentions to others, considers the worst-case scenario as inevitable or sees his/her behaviour as beyond improvement. An important point of difference between moral disengagement and self-serving cognitions is that the latter includes the primary distortions entitled “Self Centered” [20] which is a kind of psychological entitlement where one prefers one thoughts opinions and rights to others.

The existing literature reports different boundary conditions like organizational budget policy, ethical climate, employee perceptions of distributive justice, employee power distance orientation, and justice sensitivity play a contingent role in the relationship between self-serving leader behaviour and resultant different employee behaviours [15]. Recently the followers’ Machiavellianism has been found to serve as a boundary condition in the relationship between self-serving leadership and Leader-signaled knowledge hiding [21]. However, the current study took a novel approach in this regard by relying on the social learning theory to propose Machiavellianism as a boundary condition [18]in the relationship between self-serving leader behaviour and self-serving cognitive distortions. The support for this assertion comes from the literature which indicates that High Machiavellianism is characterized by a lack of empathy when manipulating others, unconcerned about the moral implications of their actions while manipulating others, a belief that the ends justify means, a pessimistic outlook about people and disdain for others, steered and supported by fraudulence, betrayal, and pursuit for benefits [22]. So, employees with such High Mach tendencies for whom the “ends justify the means”, will be more inclined to develop self-serving cognitive distortions followed by indulgence in self-serving CWBs, in presence of a self-serving leader. Moreover, such High Machs will not feel guilty to reap benefits in an unethical manner [23]. Moreover, the investigation of Machiavellianism as a trait in the current study is appropriate as Pakistan rates moderately high in both masculinity and corruption index [9]. So, such context help in the nourishment of High Mach tendencies by promoting them to target material acquisition at the cost of ethical considerations. Hence, finding High Mach tendencies in such working communities is not impossible to exist. Therefore, the third aim of the current study was to propose that in such a context high Machs will strengthen the indirect relationship between self-serving leadership and self-serving cognitive distortions through self-serving cognitive distortions.

## 2 Theory and literature review

### 2.1 Social learning theory

The link between self-serving leadership and self-serving counterproductive work behaviours through self-serving cognitions is based on the social learning theory. According to this theory, the follower learns and acquires self-serving behaviour by observing such behaviour in their leaders [16]. This theory helps understand criminal and deviant behaviours and considerable research has been done lately on deviant behaviours. In this regard, the social interactions and associations serve as a model, so the individuals imitate the observed behaviour, and in the absence of positive examples, the people adopt deviant behaviour. Moreover, the imitation of deviant behaviour comes from the observed rewards earned by the models in the course of the adoption of deviant behaviours [24]. Hence, it is proposed that the self-serving behaviour of a leader leads to self-gains, the followers observe it and create self-serving cognitions, and as a result, they adopt self-serving CWB to have similar gains for themselves.

**Fig. 1:**
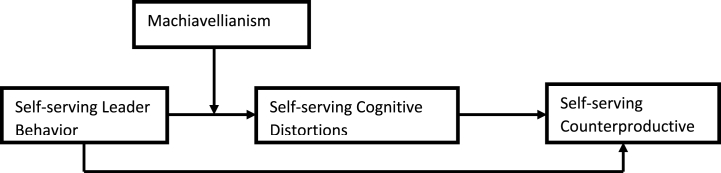
Theoretical model.

It is essential to mention that a significant tenet of social learning theory is that the existence and role of cognitions result in differences in behaviour within the same environment [25]. It means that personality differences play a role in determining the variation of behaviour among individuals in the same circumstances. Therefore, the current study proposes that employees with high Mach tendencies will be more prone to develop self-serving cognitions and indulge in self-serving CWBs in presence of a leader exhibiting self-serving behaviour. In this regard, the Fig. 1 shows the thoeretical model of the current study based on the tenets of Social Learing theory.

### 2.2 Literature review

Leader's self-serving behaviours generally impair the helping behaviour of employees [17] and boost negative work behaviours like organizational deviance, interpersonal deviance [10] and deviant work behaviours [12]. Such negative work behaviours can be attributed to the creation of an immoral work climate by a self-serving leader where the employees can place a preference for their self-interests and are not penalized for it [16]. The literature reports that such behaviour of a leader may lead to supervisor-directed deviance [2], but there is risk inherent in it. So to avoid this risk the employee directs his or her hostility toward coworkers who are low in power status [15]. Therefore, the current study proposes that self-serving counterproductive work behaviour is more likely to be exhibited. It is pertinent to mention that this is a unique form of deviant behaviour that is covert and subtle set in nature to keep one's hidden motives. In specific, it is all about furthering one's self-interest at the expense of others and is characterized by fraud and embezzlement [13].

The relationship between the self-serving leader and deviant behaviours has been explained previously through the theoretical lens of social learning theory [15]. Therefore, it is proposed that social learning [3] can provide a better explanation of the relationship between the self-serving leader and self-serving cognitive work behaviours. This theory [18] proposes that one can emulate the behaviour of a model by analyzing the consequences of his or her behaviour. Therefore, the observation of the self-serving behaviour of a leader resulting in fulfilling his or her interest in self-gain leads to the emulation of the behaviour by his or her subordinates. Therefore, the followers exhibit this behaviour and make personal gains.

H1 Leader self-serving Behaviour has a positive relationship between self-serving and counterproductive work behaviour.

Leader self-serving behaviour has been investigated with moral disengagement [17] but this study will focus on self-serving cognitions. Moreover, it is important to note that these two constructs are different as the latter is composed of primary and secondary distortions. The Primary cognitive distortions are self-centred attitudes, thoughts, and beliefs that place a high value on one's views, expectations, needs, rights, immediate feelings, and desires to the point where others' legitimate views are barely considered or ignored. The secondary distortions provide an emotional backup and reduce the stress induced by the primary distortions. It means the secondary distortions reduce the guilt and self-blame originating from primary distortions [26]. The secondary distortions include blaming others, Mislabeling, and Assuming the Worst where one considers that the worst-case scenario is the only option for exhibiting deviant behaviour and there is no room for improving one's behaviour [19].

The Social Learning Theory [18] concisely that cognitions play a role in observational learning. Therefore, it is proposed that the self-serving leader's behaviour leads to self-serving cognitions where he or she gives preference to oneself and is ready to dehumanize others, blame them, and consider that aggressive behaviour is the only option in the given circumstances.

H2 Leader self-serving behaviour has a positive relationship with self-serving cognitions

Cognitive distortions are inaccurate ways of giving meaning to different life happenings [19]. Moreover, it is important to note that the externalization of behaviours from the self-serving distortions is generally an outcome of the neutralization of compassion and guilt through processes such as misattributing blame to others or minimizing the consequences of one's antisocial actions [20]. Even, the literature reveals that self-serving cognitive distortions result in delinquent, proactive aggressive, and reactive aggressive behaviour [27], and antisocial [14,28] and deviant behaviours [18,19].

Social learning theory [18] provides the logical explanation as observational learning is cognitive and involves internal mental processing before expressing the behaviour. Therefore, the current study proposes that self-serving distortions result in self-serving counterproductive work behaviours. Moreover, it is pertinent to mention that these behaviours are covert deviant acts aimed at perusing self-interests, which may be fraudulent [13]. Likewise, these behaviours tend to be discrete, well-coordinated, and strategic which ensures that there is a benefit inherent in such acts [29].

H3 Self Serving Cognitions have a positive relationship with self-serving counterproductive work behaviour

The self-serving cognitive has been recently investigated as an underlying mechanism with individuals' maladaptive personality traits and hating behaviour [30]. Moreover, it has been studied as a mediator between callous-unemotional traits and antisocial behaviour [31]. Likewise, it has been proven to mediate the relationship between psychopathic traits and antisocial behaviours^9^. But, the study takes a novel approach and proposes that self-serving cognitive distortions serve as an underlying mechanism between a self-serving leader and self-serving counterproductive work behaviours. Even, the literature supports this assertion as it evidences that both the oppressor and his or her victims have a high level of self-serving cognitive distortions, which in turn rationalize the externalization of aggressive and anti-social behaviour [28]. Therefore, it can be inferred that the Leaders self-serving behaviour victimizes the followers and activate the self-serving cognitive distortions. These distortions as a result provide a rational justification for becoming self-serving just like their leaders. Even, the Social Learning Theory [18] supports this aforementioned proposed underlying mechanism. According to this theory, the new behaviour is learned while observing the model and processing the cognitions about the benefits, the model receives because of exhibiting any specific behaviour. It means a follower does observe that the self-serving leader's behaviour leads to personal gains for the leader. This activates the self-serving cognitive distortions that provide a rationalization for becoming self-serving to reap the same benefits that a self-serving leader is having because of his or her behaviour.

Self-serving leadership has been investigated with deviant work behaviours [12]. However, the current study proposes that the aforementioned leader behaviours activate the self-cognitive distortions that result in a specific form of counterproductive work behaviour that is self-serving. Unlike generic counterproductive work behaviours, self-serving counterproductive work behaviours are covert, strategically planned, involve hidden motives, and have a clear benefit for their perpetrator [13].

H4 Self-serving cognitive distortions mediate the relationship between a leader's self-serving behaviour and self-serving counterproductive work behaviours

Based on social learning theory, recently the followers' Machiavellianism has been proven as a disposition that strengthens the relationship between self-serving leadership and leader-signaled knowledge hiding resulting in knowledge hiding [21]. However, the current study based on social learning theory proposes that the followers' Machiavellianism will strengthen the relationship between self-serving leadership and self-serving cognitive distortions. It is important to mention that one of the central tenets of Social Learning Theory [18] is that cognitive processes, behaviour, and context interact with one another simultaneously. The aforementioned cognitive processes refer to already-held beliefs and human dispositions. Therefore, it means that cognitions or personality traits play a role in producing differences in the same context [12]. So the current study has taken up Machiavellianism as the personality trait which strengthens the indirect effect of a leader's self-serving behaviour on self-serving counterproductive work behaviours through the development of self-serving cognitive distortions. The Machs generally view that others actively seek to damage one's well-being and they have a sense of fear-based urgency to save themselves from exploitation, so the result is the cultivation of a mindset characterized by “ends justify means” [32]. So, High Machs have a general tendency to morally disengage and engage in destructive deviance [33]. Cognitive distortions are taught socially, which means they can evolve and interact with other psychological processes within the person [34,35]. Therefore, the current study proposes that high Machs in presence of a self-serving leader will be more prone to develop self-serving cognitions that result in self-serving CWBs. Moreover, when high Machs observes a self-serving leader reaping benefits in form of self-gains then High Machs emulates this behaviour as he or she can make sense that the beneficial gains outweigh the inherent risks [36].

Therefore, the current study proposes that employees with high Mach tendencies will be more prone to develop self-serving cognitions and indulge in self-serving counterproductive behaviours in presence of a leader exhibiting self-serving behaviour.

H5 Machiavellianism moderates the indirect relationship of a leader's self-serving behaviour to self-serving counterproductive work behaviours through self-serving cognitive distortions, in such a way that the relationship becomes stronger at higher levels.

## 3 Methodology

The FMS Ethics Research Board of International Islamic University Islamabad-IIUI has granted the ethical approval for this study with approval number FMS-HRM 32–2022. So after approval the data collection for the present study was done by circulation of close-ended questionnaires to the researcher's contacts. The data was collected from the service sector organizations operating in the Twin cities of Islamabad and Rawalpindi, Pakistan. The data was collected from the service sector organizations (both public and private sector banks, educational institutions, telecom companies and marketing agencies) operating in the Twin cities of Islamabad and Rawalpindi, Pakistan. The rationale behind such a diverse sample was to make enhance the generalizability [61] of the current study operating in the service sector.

In this regard, it is important to note that for each organization a particular contact person was identified who obtained verbal consent from the sample of employees, followed by circulation and collection of these questionnaires distributed in three-time lags. At Time 1, the participants were selected based on convenience sampling which lead to self-selection in the latter time lags by getting responses from those who already responded in the following time lags. The matching of questionnaires for the three-time lags was done with a unique identification code composed of the first alphabet of first and last name followed by birth month mentioned in numeric form. At Time 1, Self-serving leadership behaviour and Machiavellianism were measured, during this time lag 450 questionnaires were distributed but 425 questionnaires were received back. At Time 2 Self-serving cognitive distortions were measured and during this time lag 425 questionnaires were distributed but 399 were received back. At Time 3 self-serving counterproductive work behaviour was measured, and during this lag, 399 questionnaires were distributed and 383 were received back. After three times matching and discarding the incomplete questionnaires, the analysis was done on 375 cases.

The data that support the findings is available in Open Science Framework at https://osf.io/jwehs/?view_only=a55d6b56195145afa85c9912bac9e628 following an embargo from the date of publication to allow for the commercialization of research findings.

The study sample was composed of more males (54.9%) than females (45.1%), with a majority of them being single (64.8%). The majority of the sample (86.1%) had an age range of 26–35 years. Some of the respondents had bachelor's (34.1%) while others had master's degrees (40.3%). The vast majority had a total working experience of 2–5 years. Moreover, the majority (90.9%) had an experience of more than one year with an existing supervisor. The sample was composed of employees working in government and semi-government (54%), and private organizations (46%) located in Rawalpindi and Islamabad.

### 3.1 Measures

#### 3.1.1 Self-serving leader behaviour

This construct was measured using the four-item scale of Self serving leadership [3] with five points Likert scale ranging from 1 as strongly disagree to 5 strongly agree. Sample items are “My supervisor is selfish and thinks he/she is very important” and “My supervisor uses resources of the company for him/herself”. This is a short scale for measuring self-serving leadership and has been validated and utilized in multiple studies to assess self-serving leadership^15,16^.

The current study revealed that single-factor CFA results showed that the model fits well (Χ^2^ = 12.982, DF = 5, Χ^2^/DF = 2.596, CFI = 0.992, NFI = 0.987, GFI = 0.986, TLI = 0.984, RMR = 0.034, RMSEA = 0.065).

#### 3.1.2 Self-serving cognitive distortions

The original scale is of 54 items, but only 39 items were utilized from The How I Think Questionnaire [19] having four dimensions which include self-centred, blaming others, minimizing/mislabeling, and assuming the worst. This rating scale was evaluated on 6 points Likert scale varying from 1 as strongly disagree to 6 strongly agree. The sample item is “If I really want to do something, I don't care if it is legal or not.”

The current HIT questionnaire has been validated and used to measure self-serving cognitive distortions by different studies [30,31,[37], [38], [39]].

Furthermore, the second-order CFA of self-serving cognitive distortions indicated a good fit (Χ^2^ = 1424.54, DF = 676, Χ^2^/DF = 2.107, CFI = 0.937, NFI = 0.887, GFI = 0.834, TLI = 0.931, RMR = 0.176, RMSEA = 0.054) as compared to four-dimensional CFA results (Χ^2^ = 3465.914, DF = 696, Χ^2^/DF = 4.980, CFI = 0.767, NFI = 0.726, GFI = 0.668, TLI = 0.752, RMR = 0.199, RMSEA = 0.103).

#### 3.1.3 Machiavellianism

This construct was measured with twelve item scale on Machiavellianism [32] rated on 5 points Likert scale varying from 1 as strongly disagree to 5 strongly agree. When compared to two dimensional CFA results (Χ^2^ = 161.146, DF = 53, Χ^2^/DF = 3.040, CFI = 0.927, NFI = 0.898, GFI = 0.931, TLI = 0.911, RMR = 0.102, RMSEA = 0.074), a 2nd order CFA indicated a better fit (Χ^2^ = 55.167, DF = 46, Χ^2^/DF = 1.199, RMR = 0.056, GFI = 0.977, CFI = 0.994, NFI = 0.965, TLI = 0.911, RMSEA = 0.023).

Table 1 illustrates the model fit indices of the confirmatory factor analysis to establish convergent and discriminant validity. Thorough scrutiny of the model fit indices showed that at time 1 a two-factor model showed a better fit as compared to the one-factor model. The results showed that the four-factor model had a better model fit (Χ^2^ = 2726.63, DF = 1839, Χ^2^/DF = 1.48, CFI = 0.95, NFI = 0.0.85, GFI = 0.82, TLI = 0.94, RMR = 0.12, and RMSEA = 0.03) than a single factor.

**Table 1:** Confirmatory factor analysis for study variables.

Measurement Model	Χ^2^	DF	Χ^2^/DF	CFI	NFI	GFI	TLI	RMR	RMSEA
1. LSSB and Mach (2 Factor) Time 1	237.41	116	2.05	.92	.91	.93	.94	.08	.05
LSSB and Mach (1 Factor) Time 1	387.70	118	3.3	.89	.85	.896	.87	.23	.08
2. Full Measurement Model (4 Factor)	2726.63	1839	1.48	.95	.85	.82	.94	.12	.03
3. Full Measurement Model (1 Factor)	9251.91	1952	4.74	.54	.49	.47	.53	.16	.10

Note: LSSB = Leader Self serving Behaviour, Mach = Machiavellianism, SSCD (Self-serving Cognitive Distortions) and SSCWB (Self-serving Counterproductive work behaviour).

#### 3.1.4 Self-serving counterproductive work behaviour

This construct was measured with 8 items Self-Serving Counterproductive Work Behaviour scale [13]. Its response was recorded on a 6-point Likert scale (1 as strongly disagree to 6 as strongly agree). It is a validated measure and has been recently utilized in another study [40]. In the current study, a single factor confirmatory factor analysis shows that the model fits well, with Χ^2^ = 33.708, DF = 12, Χ^2^/DF = 2.809, CFI = 0.976, NFI = 0.964, GFI = 0.978, TLI = 0.944, RMR = 0.086, RMSEA = 0.070.

## 4 Results

A theoretical and empirical overlap between moral disengagement and self-serving cognitive distortions intending to develop a questionnaire of moral neutralization [41]. However, a noteworthy fact is that they eliminated the primary distortion and included only secondary distortions. This meant that of the four dimensions, they analyzed three dimensions. Therefore, the current study did an empirical investigation in this regard. Firstly, the bivariate correlation revealed a very weak positive correlation (*r* = .12, *p* < 0 0.05) between the construct of self-serving cognitive distortions composed of four dimensions and moral disengagement. Secondly, a two-factor CFA model that loads the two constructs on separate latent variables (Χ^2^ = 470, DF = 167, Χ^2^/DF = 2.7, CFI = 0.95, NFI = 0.91, GFI = 0.84, TLI = 0.95, RMR = 0.084, RMSEA = 0.07) had a better fit than the one factor that forces all items to be loaded onto a single latent variable (Χ^2^ = 5924.753, DF = 1034, Χ^2^/DF = 5.73, CFI = 0.61, NFI = 0.57, GFI = 0.49, TLI = 0.59, RMR = 0.17, RMSEA = 0.112).

Table 2 illustrates that the convergent validity of all study variables is established as the CR is above 0.7 and AVE is above 0.5 [42]. Discriminant validity is as well established as AVE > MSV for all study variables [43]. Moreover, there is no evidence of multicollinearity as the leader's self-serving behaviour has a weak positive correlation (r = 0.2, p < 0.01) with the moderator Machiavellianism. Moreover, a leader's self-serving behaviour has a positive association with self-serving cognitive distortions (r = 0.32, p < 0.01) and self-serving counterproductive work behaviours (r = 0.30, p < 0.01). Furthermore, the self-serving cognitive distortions had a positive association with self-serving counterproductive work behaviour (r = 0.50, p < 0.01). In addition to these results, the Self-serving cognitive distortions had a very weak positive relationship with moral disengagement.

**Table 2:** Convergent & Discriminant validities; Mean, Standard Deviation and Bivariate correlations of Study variables.

Sr		CR	AVE	MSV	Mean	SD	Age	Dep	LSSB	Mach	SSCD	SSCWB	
1	Age				2.09	.38							
2	Dep				4.31	2.06	−.02						
3	LSSB	0.87	0.64	0.09	3.33	1.12	−.02	−.00	(.891)				
4	Mach	0.77	0.65	0.45	3.23	.70	.02	−.12*	.20**	(.965)			
5	SSCD	0.91	0.74	0.63	2.83	.90	−.09	−.03	.32**	.600**	(.804)		
6	SSCWB	0.78	0.56	0.24	3.33	.88	−.16**	−.12*	.30**	.330**	.501**	(.70)	

Note: N = 375; Cronbach alpha reliabilities are shown in parenthesis.*p < 0.05, **p < 0.01. LSSB = Leader Self-serving Behaviour, Mach = Machiavellianism, SSCD= Self-serving Cognitive Distortions, SSCWB=Self-serving CWB.

Table 3 shows that the H1 was supported as the direct effect of self-serving leadership on self-serving CWB (b = 0.13, p < 0.001). Moreover, H2 was supported as self-serving leader behaviour has a significant effect on self-serving cognitive distortions (b = 0.18, p < 0.001). Moreover, H3 was supported as the direct effect of self-serving cognitive distortions on self-serving counterproductive work behaviours (b = 0.42, p < 0.01).

**Table 3:** Moderated mediation results.

	SSCD				SSCWB			
	Beta	SE	LLCI	ULCI	Beta	SE	LLCI	ULCI
Constant	3.21***	0.21	2.79	3.63	3.93***	0.27	2.3	3.47
Age	−0.22*	0.09	−0.40	−0.04	−0.28**	0.10	−0.48	−0.08
Dept	0.01	0.01	−0.01	0.05	−0.05**	0.18	−0.08	−0.01
LSSB	0.18***	0.03	0.11	0.24	−0.13***	0.04	0.05	0.19
SSCD					0.42**	0.33	0.13	0.51
Mach	0.69***	0.05	0.58	0.79				
LSSB × Mach	0.17*	0.05	0.07	0.26				
R^2^	0.43					0.30		
ΔR^2^	0.0182***							
Moderator-Machiavellianism					LSSB→SSCD→SSCWB			
					Effect	SE	LLCI	ULCI
Conditional indirect Effects			(-1)SD		0.02	0.01	−0.00	0.05
			(+1)SD		0.13	0.02	0.07	0.18
Index of moderated Mediation					0.07	0.02	0.02	0.11

Note: Sample size = 375. *p < 0.05, **p < 0.01, ***P < 0.001. LSSB = Leader Self-serving Behaviour, Mach = Machiavellianism, SSCD= Self-serving Cognitive Distortions, SSCWB=Self-serving CWB.

The H4, that is, the mediation hypothesis was tested with process macro 4 proposed by Hayes (2013). The output revealed a significant indirect effect (b = 0.12, SE = 0.02, 95% CI = 0.07, 0.15). Hence H4 was supported empirically signifying the existence of an underlying mechanism of self-serving cognitive distortions between self-serving leader behaviour and self-serving counterproductive work behaviours (see Fig. 1).

**Fig. 2:**
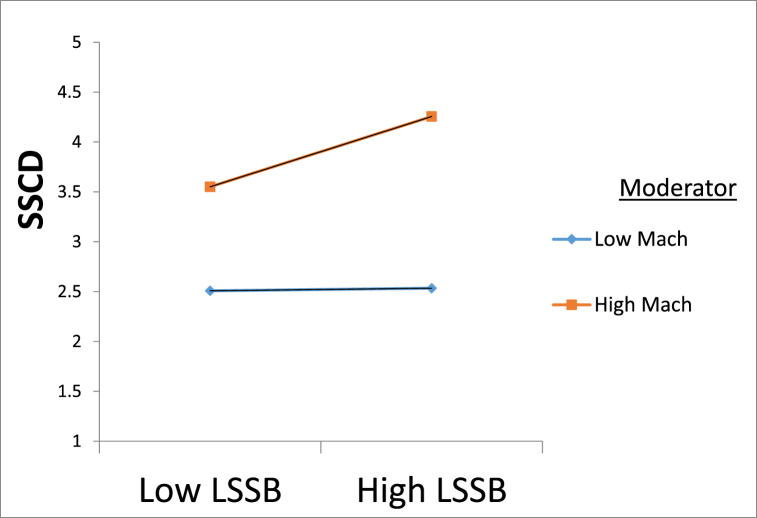
Moderating role of Machivellianism in Relationship between Leader self serving behaviour and Self serving cognitive distortions.

The moderated mediation was carried out with model 7 proposed by Hayes (2013) instead of Structural Equation Modeling (SEM) as the latter does not offer the slope test. In this regard, output in Table 3 revealed Machiavellianism strengthened the relationship between leaders' self-serving behaviour and self-serving cognitive distortions (β = 0.17, p < 0.001, CI [0.07, 0.26], ΔR ^2^ = 0.0182). Furthermore, the slope test implies at −1 SD, the ß = 0.06, p > 0.01, whereas at +1 SD the beta got reduced to ß = 0.30, p < 0.001. This signifies that High Mach strengthens the positive relationship between a leader's self-serving behaviour and self-serving cognitive distortions. In this regard, the graphical presentation (Fig. 2) reveals that at high levels of Machiavellianism, the slope becomes more positively slanted as compared to a weak but flatter positive slope between leader self-serving behaviour and self-serving cognitive distortions, at low levels of Machiavellianism. The conditional indirect effect of leader self-serving behaviour and self-serving counterproductive behaviour through self-serving cognitive distortions, at a high level of Mach (ß = 0.13, CI = 0.07,0.18) was significant and greater than at a low level of Mach. Moreover, the index of moderated mediation (ß = 0.07, CI = 0.02, 0.11) as well confirmed that at a High level of Mach, the conditional indirect effect of leader self-serving behaviour to self-serving counterproductive work behaviour through self-serving cognitive distortions strengthened. Hence, H5 was as well supported.

## 5 Discussion

The aim of the current study was three-fold. The first aim was achieved by testing H1 that empirically established a positive relationship between leaders' self-serving behaviour and self-serving counterproductive work behaviour. In this regard it is evident that leader self-serving behaviour, in general, has been investigated with different behaviours like organization citizenship behaviour, helping behaviour, knowledge hiding, supervisor-directed deviance, and deviant work behaviours [12,16,17,44]. So in the light of existing literature, the current study proposed and empirically established a positive relationship with self-serving counterproductive work behaviours. It is pertinent to note that self-serving leadership only looks out for its interests, and its deprivation of and harm to subordinates are relatively more covert [23]. This is primarily manifested in threats to the interests of subordinates or teams, which frequently results in a lot of unspoken pressure and insecurity on the part of the subordinates. This contrasts with the hostile and combative behaviours displayed by other negative leaders (e.g abusive supervision) [45]. When under a lot of pressure, a subordinate could act in ways that are counter to the objectives of the organization and its employees.

The second aim was achieved by empirically testing the H2 and H3 and followed by empirical investigation of the H4 proposing an underlying mechanism of self-serving cognitive distortions in the relationship between leaders' self-serving behaviour and self-serving counterproductive work behaviour. In this regard, the literature reveals that moral disengagement has been investigated as an underlying mechanism between leaders' self-serving behaviour and helping behaviour [17]. It is pertinent to note that moral disengagement denotes a set of cognitive strategies individuals use to evade ethical self-regulatory processes that generally avoid transgression [6]. On the other hand, self-serving cognitive distortions are composed of primary and secondary distortions [19]Primary cognitive distortions are self-centred attitudes, thoughts, and beliefs that place a high value on one's views, expectations, needs, rights, immediate feelings, and desires to the point where others' legitimate views are barely considered or ignored. The secondary distortions provide an emotional backup and reduce the stress induced by the primary distortions. These secondary distortions resemble the cognitive processes of moral disengagement [41]. However, an important consideration is that the current study took both primary and secondary distortions as a second-order latent variable entitled self-serving cognitive distortions. The existing literature supports this higher-order latent construct as a mediator [28,30,31]. Moreover, the detailed empirical investigation of the current study proved moral disengagement and self-serving cognitive distortions as two distinct constructs. Therefore, the current study took a novel approach by utilizing the aforementioned self-serving cognitive distortions as an underlying mechanism between the leader's self-serving behaviour and self-serving counterproductive work behaviours.

The third aim of the current study was the empirical establishment of High Machiavellian Tendencies strengthening the indirect relationship between leaders' self-serving behaviour to self-serving counterproductive work behaviour through self-serving cognitive distortions. The current literature indicates that Machiavellianism plays a prominent role in determining the dynamics of the leader and follower relationship [46]. It is noteworthy that high Machs value the prospects which lead to personal rewards [47]. So it is understandable that a high Mach will alter his or her behaviour by observing the benefits gained by a self-serving leader and will indulge in self-serving counterproductive work behaviours. The existing literature as well supports this tendency of high Machs to indulge in different counterproductive work behaviours [48,49] and deviant behaviour [50]. Moreover, this deployment of unethical strategies by High Machs can be explained by several mechanisms. First, a sceptical and destructive worldview leads to expecting the worst from others. Second, egoism and a strong focus on High Machs lead to a lack of commitment and attachment to the employees and organizations. Third, a lack of emotional attachment results in zero guilt because of engagement in unethical behaviour [51]. So it was proposed with the theoretical lens of social learning theory [18] that High Mach tendencies will expedite the indirect relationship between a leader's self-serving behaviour to self-serving CWB through self-serving cognitive distortions.

### 5.1 Theoretical implications

Self-serving leader behaviour has been investigated with negative work-related behaviours like knowledge hiding, supervisor-directed deviance, deviant work behaviours, organizational deviance, and interpersonal deviance [12,15,16,44]. However, the current study took self-serving counterproductive behaviour which is a specific form of covert behaviour intended to benefit oneself and is characterized by fraud and embezzlement [13]. But to the best of researchers' knowledge, it has not been investigated with self-serving counterproductive work behaviours. Moreover, this study fills the gap in the literature [13] which specifies that leadership can play a role in instigating self-serving CWBs.

The proposed mechanism of self-serving cognitive distortions between the Leader's self-serving behaviour and counterproductive work behaviours is unique. The current literature has investigated the self-serving cognitive distortions between negative personality traits like maladaptive traits, and callous-unemotional traits, and negative work behaviours like hating and antisocial behaviour [30,31]. Not only do such negative dispositions but the context (Bullying) as well play a role in the activation of self-serving cognitive distortions resulting in antisocial behaviours [52]. But to the best of researchers' knowledge, this underlying mechanism has not been investigated with dark leadership styles. So the current study has taken a novel approach by proposing that self-serving leadership activates the self-serving cognitive distortions that result in self-serving CWB. The leader's self-serving behaviour has been investigated through the theoretical lens of Social learning theory but with different underlying mechanisms like psychological safety and team hiding [16], and moral disengagement [17]. But the current study takes a novel approach by utilizing the underlying mechanism of self-serving cognitive distortions in the light of social learning theory.

The existing literature depicts that followers' Machiavellianism strengthens the indirect relationship between ethical leadership style with corruption through intuitive thinking [53]. More specifically, recently the followers’ Machiavellianism has been found to serve as a boundary condition in the relationship between self-serving leadership and Leader-signaled knowledge hiding [21] But in contrast, the current study has established that High Mach tendencies strengthen the positive indirect effect of self-serving leadership on self-serving counterproductive work behaviour through self-serving cognitive distortions. So, this study adds to the literature of Social learning theory by investigating the High Mach tendencies as the personality trait that enhances the indulgence in self-serving counter productive work behaviors through activation of self serving cognitive distortions in presence of a self-serving leader.

### 5.2 Practical implications

It is very common for leaders to act in self-serving ways but organizations need to create a climate that emphasizes “a focus on others” to shift the emphasis from personal goals to collective gains for everyone. In such a climate, the leaders will be more prone to take into account the collective interest instead of opting for self-gains [23]. In addition, it is advised to organizations for having strict monitoring systems so that both the leaders and followers act according to the larger collective interests rather than for self-serving motives while taking different decisions [54]. It is pertinent to note with such strict monitoring systems along with the “focus on others” adopted by the leaders will have a trickle-down effect on the subordinates who will emulate their leaders' behaviours and in such circumstances, the self-serving cognitive distortions may not get activated.

In addition, organizations should make more strict scrutiny about selection criteria for hiring managers or supervisors. For example avoid hiring candidates having narcissistic tendencies as such individuals are more to indulge in CWBs [55]. Moreover, screen the candidates who show guilt for indulgence in self-interest behaviours and with high levels of Honesty-humility as this tendency negatively predicts unethical acts [23]. Moreover, it is suggested to give promotion to the supervisory or managerial role to the communal-oriented rather than the exchange-oriented candidates. The reason is that the latter type of candidate is reported to indulge in self-interested behaviours [56].

The self-serving tendencies of existing leaders can be reduced by educating them to see the world from their follower's perspective that taking into account their interests. Such interventions will help the leaders develop a new schema [57]. So, organizations can develop training programs to help leaders comprehend the detrimental effects that self-serving leadership can have on their employees.

If self-serving leadership offers a challenge to organizations then managers should hire employees with Low Mach Tendencies. As low Machs will develop guilt because of engaging in unethical acts [51]. So it can be inferred that such employees will low Mach tendencies will not activate the self-serving cognitions and hence will not indulge in self-serving CWBs.

## 6 Limitations, strengths, and future research directions

A basic limitation of the current study is that the data was collected from service sector companies located in the Twin cities of Rawalpindi and Islamabad, Pakistan. Still, the generalizability can be improved in the future by analyzing the proposed model for both the service and manufacturing sectors.

The basic strength of the current study was that the data was collected in three-time lags with self-reported collected for Self-serving cognitive distortions and Machiavellianism and peer-reported data for self-serving counterproductive work behaviour. Literature supports the utilization of self-reports as it can be the most appropriate method to get the true picture [58]. On the other hand, peer reporting reduces the common method bias and social desirability concerns [59]. Moreover, the study design is time-lagged which leads to self-selection at Time 2 and Time 3 almost addresses the common method bias issue.

It is suggested to utilize some contextual variables like self-interest climate in contrast to Machiavellianism trait for determining the indirect effect of self-serving leadership on self-serving counterproductive work behaviours through self-serving cognitive distortions. Moreover, the literature as well supports that the self-interest climate strengthens unethical behaviours [60].

## 7 Conclusion

The current study was a unique investigation of Self Serving Leadership in Pakistani service sector organizations operating in a cultural context characterized by High power distance, high uncertainty avoidance and moderately high levels of masculinity. The current study added to the limited literature on the Dark side of Leadership dynamics which is still underexplored in the Asian region. Moreover, this study added to the literature of Social learning theory by utilizing self-serving cognitive distortions as an underlying mechanism between the self-serving leader behaviour and self-serving CWB. The current study proposed and empirically proved that self-serving cognitive distortions are distinct from moral disengagement. Moreover, there is limited literature on the utilization of self-serving cognitive distortions as an underlying mechanism in organization-based literature. Moreover, the current study empirically established that in presence of high Mach tendencies, the self-serving cognitive distortions are activated and followers are prone to indulge in self-serving CWB.

## Author contribution statement

1. Uzma Sarwar: conceived and designed the experiments; analyzed and interpreted the data; contributed reagents, materials, analysis tools or data; wrote the paper.

2. Sidrah Al Hassan: conceived and designed the experiments; analyzed and interpreted the data; wrote the paper.

3. Osama Khassawneh: analyzed and interpreted the data; wrote the paper.

4. Tamara Mohammad: analyzed and interpreted the data; wrote the paper.

5. Rashida Parveen: analyzed and interpreted the data; contributed reagents, materials, analysis tools or data.

## Funding statement

This research did not receive any specific grant from funding agencies in the public, commercial, or not-for-profit sectors.

## Data availability statement

Data associated with this study has been deposited at https://osf.io/jwehs/?view_only=a55d6b56195145afa85c9912bac9e628.

## Additional information

Supplementary content related to this article has been published online at [URL].

## Declaration of competing interest

The authors declare that they have no known competing financial interests or personal relationships that could have appeared to influence the work reported in this paper.
