# Corrigendum to “One pot sets another boiling: A case of social learning perspective about leader self-serving behaviour and followers self-serving counterproductive work behaviour” [Heliyon **9** (3), March 2023, Article e14611]

**DOI:** 10.1016/j.heliyon.2023.e15216

**Published:** 2023-04-05

**Authors:** Uzma Sarwar, Sidrah Al Hassan, Osama Khassawneh, Tamara Mohammad, Rashida Parveen

**Affiliations:** a School of Education, Huanggang Normal University, Huanggang, 438000, PR China; b Department of Business Administration, Faculty of Management Sciences, International Islamic University Islamabad (IIUI), Pakistan; c The Emirates Academy of Hospitality Management, United Arab Emirates; d American University in the Emirates, Dubai, United Arab Emirates

In the original published version of this article, one of the co-authors, Uzma Sarwar, has requested to change the corresponding authorship to first author. This now has been corrected.

The authors apologize for this error. Both the HTML and PDF versions of the article have been updated to correct the error.

## Declaration of interest’s statement

The authors declare no conflict of interest.

