# Supplementary material for: One pot sets another boiling: A case of social learning perspective about leader self-serving behaviour and followers self-serving counterproductive work behaviour
Source: Heliyon. 2023 Mar 18;9(3):e14611. doi: 10.1016/j.heliyon.2023.e14611 (PMC10040702; doi:10.1016/j.heliyon.2023.e14611)
Supplement: Multimedia component 1 [file mmc1.docx]

**Questionnaire**

**Time 1**

**PART A: pERSONAL INFORMATION**


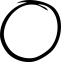


Please circle the appropriate number for your response. **Date: / /** QID:______________

Time: T1

| **1.** | Please write the initials of your first and last name followed by your birth month  (e.g. Majid Khan born in July, M.K.07) | | | | |  |  |  | |  |
| --- | --- | --- | --- | --- | --- | --- | --- | --- | --- | --- |
|  | | | | | | | | | | |
| **2.** | **Email / Mobile** | |  | | | | | | | |
|  | | | | | | | | | | |
| **3.** | **Gender** | | Male | 1 | Female | | | | 2 | |
|  | | | | | | | | | | |
| **4.** | | **Marital status** | single | 1 | married | | | | 2 | |
|  |  |  | divorce | 3 | widowed | | | | 4 | |
|  | |  |  |  |  |  |  |  |  |  |
| **5.** | | **Age Group** | Below 25 | 1 | 26 – 35 years | | | | 2 | |
|  |  |  | 36 - 45 years | 3 | 46 – 55 years | | | | 4 | |
|  |  |  | 56 and above | 5 |  | | | |  | |
|  | |  |  |  |  | | | |  | |
| **6.** | | **Educational qualification** | Bachelors | 1 | Masters | | | | 2 | |
|  |  |  | MS/ M.Phil | 3 | Ph.D. | | | | 4 | |
|  |  |  | Post Doctorate | 5 |  | | | |  | |
|  | | | | | | | | | | |
| **7.** | | **Area of specialization** | HR | 1 | Marketing | | | | 2 | |
|  |  |  | Banking & Finance | 3 | IT and Software | | | | 4 | |
|  |  |  | Electric/Civil Engineering | 5 | Medical sciences | | | | 6 | |
|  |  |  | Law and taxation | 7 | Project Management | | | | 8 | |
|  |  |  | Others (please specify) | 9 |  | | | | | |
|  | |  |  |  |  | | | |  | |
| **8.** | | **Type of organization** | Government | 1 | semi government | | | | 2 | |
|  |  |  | private | 3 | MNC | | | | 4 | |
|  |  |  | Other(please specify)_____________________________________ | | | | | | | |
|  | |  |  | | | | | | | |
| **9.** | | **Name of your organization** |  | | | | | | | |
|  | | | | | | | | | | |
| **10.** | | **Your designation/grade**  **(Specify appropriate or equivalent)** | Chairman/GM | 1 | Director | | | | 2 | |
|  |  |  | Dy. Director | 3 | Assistant Director | | | | 4 | |
|  |  |  | Sr Manager/Manager | 5 | Assistant Manager | | | | 6 | |
|  |  |  | Executive | 7 | Officer | | | | 8 | |
|  | |  |  |  |  | | | |  | |
| **11.** | | **Department you are currently working in** | Admin | 1 | HR | | | | 2 | |
|  |  |  | Marketing | 3 | Finance | | | | 4 | |
|  |  |  | IT | 5 | Procurement | | | | 6 | |
|  |  |  | Production | 7 | R & D | | | | 8 | |
|  |  |  | Other(please specify)_____________________________________ | | | | | | | |
|  | |  |  |  |  | | | |  | |
| **12.** | | **Total Working Experience** | Less than 2 Years | 1 | 2 to 5 years | | | | 2 | |
|  |  |  | 6 to 10 years | 3 | More then 10 years | | | | 4 | |
|  | | | | | | | | | | |
| **13.** | | **How long have you been working with your present company** | Less than 2 Years | 1 | 2 to 5 years | | | | 2 | |
|  |  |  | 6 to 10 years | 3 | More then 10 years | | | | 4 | |

| **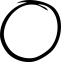**  Please circle the appropriate number for your response | Strongly Disagree | Disagree | Neutral | Agree | Strongly Agree |
| --- | --- | --- | --- | --- | --- |
|  | | | | | |
| **Does your Boss act in a following way?**  **Leader Self-serving behavior-Camps, Decoster, and Stouten (2012)** | | | | | |
| 1. My supervisor would forge a document when this would improve his/her position | 1 | 2 | 3 | 4 | 5 |
| 1. My supervisor is selfish and thinks he/she is very important | 1 | 2 | 3 | 4 | 5 |
| 1. My supervisor does not show consideration for his/her followers, only for him/herself | 1 | 2 | 3 | 4 | 5 |
| 1. My supervisor uses resources for the company for his/herself | 1 | 2 | 3 | 4 | 5 |
| **To what degree you agree or disagree with the following statements?**  **Machevellianism-Monaghan et al., (2019)** | | | | | |
| 1. In my opinion, human nature is to be dishonest | 1 | 2 | 3 | 4 | 5 |
| 1. I think that most people will take advantage of others in the right situation | 1 | 2 | 3 | 4 | 5 |
| 1. When people do something nice for me they really have another agenda | 1 | 2 | 3 | 4 | 5 |
| 1. I feel that deep down people trust each other | 1 | 2 | 3 | 4 | 5 |
| 1. I think people would rather help each other than act selfishly | 1 | 2 | 3 | 4 | 5 |
| 1. I believe that most people are essentially good | 1 | 2 | 3 | 4 | 5 |
| 1. I think that it is OK to be unethical for the greater good | 1 | 2 | 3 | 4 | 5 |
| 1. I think that it is OK to take advantage of others to achieve an important goal | 1 | 2 | 3 | 4 | 5 |
| 1. It is sometimes necessary for me to mislead others to get things done | 1 | 2 | 3 | 4 | 5 |
| 1. I value being honest over getting ahead | 1 | 2 | 3 | 4 | 5 |
| 1. To me, it is never justified to deceive others | 1 | 2 | 3 | 4 | 5 |
| 1. To me, something is not worth doing if it requires being unethical | 1 | 2 | 3 | 4 | 5 |
| 1. In my department people’s primary concern is their personal benefits | 1 | 2 | 3 | 4 | 5 |
| 1. People in my department think of their own welfare first when faced with difficult decision | 1 | 2 | 3 | 4 | 5 |
| 1. People in my department are very concerned about what is best for them personally | 1 | 2 | 3 | 4 | 5 |
| 1. People around here protect their own interest above other considerations | 1 | 2 | 3 | 4 | 5 |
| 1. People around here are mostly out for themselves | 1 | 2 | 3 | 4 | 5 |

**Time 2**

**PART A: pERSONAL INFORMATION**


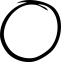
Please circle the appropriate number for your response. **Date: / /**  QID:______________

Time: T2

| **1.** | Please write the initials of your first and last name followed by your birth month  (e.g. Majid Khan born in July, M.K.07) | | | | |  |  |  | |  |
| --- | --- | --- | --- | --- | --- | --- | --- | --- | --- | --- |
|  | | | | | | | | | | |
| **2.** | **Email / Mobile** | |  | | | | | | | |
|  | | | | | | | | | | |
| **3.** | **Gender** | | Male | 1 | Female | | | | 2 | |
|  | | | | | | | | | | |
| **4.** | | **Marital status** | single | 1 | married | | | | 2 | |
|  |  |  | divorce | 3 | widowed | | | | 4 | |
|  | |  |  |  |  |  |  |  |  |  |
| **5.** | | **Age Group** | Below 25 | 1 | 26 – 35 years | | | | 2 | |
|  |  |  | 36 - 45 years | 3 | 46 – 55 years | | | | 4 | |
|  |  |  | 56 and above | 5 |  | | | |  | |
|  | |  |  |  |  | | | |  | |
| **6.** | | **Educational qualification** | Bachelors | 1 | Masters | | | | 2 | |
|  |  |  | MS/ M.Phil | 3 | Ph.D. | | | | 4 | |
|  |  |  | Post Doctorate | 5 |  | | | |  | |
|  | | | | | | | | | | |
| **7.** | | **Area of specialization** | HR | 1 | Marketing | | | | 2 | |
|  |  |  | Banking & Finance | 3 | IT and Software | | | | 4 | |
|  |  |  | Electric/Civil Engineering | 5 | Medical sciences | | | | 6 | |
|  |  |  | Law and taxation | 7 | Project Management | | | | 8 | |
|  |  |  | Others (please specify) | 9 |  | | | | | |
|  | |  |  |  |  | | | |  | |
| **8.** | | **Type of organization** | Government | 1 | semi government | | | | 2 | |
|  |  |  | private | 3 | MNC | | | | 4 | |
|  |  |  | Other(please specify)_____________________________________ | | | | | | | |
|  | |  |  | | | | | | | |
| **9.** | | **Name of your organization** |  | | | | | | | |
|  | | | | | | | | | | |
| **10.** | | **Your designation/grade**  **(Specify appropriate or equivalent)** | Chairman/GM | 1 | Director | | | | 2 | |
|  |  |  | Dy. Director | 3 | Assistant Director | | | | 4 | |
|  |  |  | Sr Manager/Manager | 5 | Assistant Manager | | | | 6 | |
|  |  |  | Executive | 7 | Officer | | | | 8 | |
|  | |  |  |  |  | | | |  | |
| **11.** | | **Department you are currently working in** | Admin | 1 | HR | | | | 2 | |
|  |  |  | Marketing | 3 | Finance | | | | 4 | |
|  |  |  | IT | 5 | Procurement | | | | 6 | |
|  |  |  | Production | 7 | R & D | | | | 8 | |
|  |  |  | Other(please specify)_____________________________________ | | | | | | | |
|  | |  |  |  |  | | | |  | |
| **12.** | | **Total Working Experience** | Less than 2 Years | 1 | 2 to 5 years | | | | 2 | |
|  |  |  | 6 to 10 years | 3 | More then 10 years | | | | 4 | |
|  | | | | | | | | | | |
| **13.** | | **How long have you been working with your present company** | Less than 2 Years | 1 | 2 to 5 years | | | | 2 | |
|  |  |  | 6 to 10 years | 3 | More then 10 years | | | | 4 | |

| 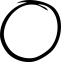  Please circle the appropriate number for your response | Strongly Disagree | Disagree | Slightly Disagree | Slightly Agree | Agree | Strongly Agree |
| --- | --- | --- | --- | --- | --- | --- |
| **The How I Think Questionnaire (Barriga et al., 2001)** | | | | |  | |
| 1. Sometimes you have to lie to get what you want. | 1 | 2 | 3 | 4 | 5 | 6 |
| 1. If I see something I like, I take it. | 1 | 2 | 3 | 4 | 5 | 6 |
| 1. When I get mad, I don’t care who get hurt. | 1 | 2 | 3 | 4 | 5 | 6 |
| 1. If I really want something, it doesn’t matter how I get it. | 1 | 2 | 3 | 4 | 5 | 6 |
| 1. You should get what you need, even if it means someone has to get hurt. | 1 | 2 | 3 | 4 | 5 | 6 |
| 1. Rules are mostly meant for other people. | 1 | 2 | 3 | 4 | 5 | 6 |
| 1. Getting what you need is the only important thing. | 1 | 2 | 3 | 4 | 5 | 6 |
| 1. If I lied to someone, that’s my business | 1 | 2 | 3 | 4 | 5 | 6 |
| 1. If I really want to do something, I don’t care if it’s legal or not. | 1 | 2 | 3 | 4 | 5 | 6 |
| 1. If I made a mistake, it’s because I got mixed up with the wrong crowd | 1 | 2 | 3 | 4 | 5 | 6 |
| 1. If someone leaves a car unlocked, they are asking to have it stolen. | 1 | 2 | 3 | 4 | 5 | 6 |
| 1. It’s OK to tell a lie if someone is dumb enough to fall for it. | 1 | 2 | 3 | 4 | 5 | 6 |
| 1. If a store or a home owner gets robbed, it’s really their fault for not having better security. | 1 | 2 | 3 | 4 | 5 | 6 |
| 1. People force you to lie if they ask too many questions. | 1 | 2 | 3 | 4 | 5 | 6 |
| 1. People are always trying to start fights with me. | 1 | 2 | 3 | 4 | 5 | 6 |
| 1. If someone is careless enough to lose a wallet, they deserve to have it stolen. | 1 | 2 | 3 | 4 | 5 | 6 |
| 1. If people don’t cooperate with me, it’s not my fault if someone gets hurt. | 1 | 2 | 3 | 4 | 5 | 6 |
| 1. When I lose my temper, it’s because people try to make me mad. | 1 | 2 | 3 | 4 | 5 | 6 |
| 1. Sometimes you have to hurt someone if you have a problem with them. | 1 | 2 | 3 | 4 | 5 | 6 |
| 1. People need to be roughed up once in a while. | 1 | 2 | 3 | 4 | 5 | 6 |
| 1. You have to get even with people who don’t show you respect. | 1 | 2 | 3 | 4 | 5 | 6 |
| 1. Everybody lies, it’s no big deal. | 1 | 2 | 3 | 4 | 5 | 6 |
| 1. If you know you can get away with it, only a fool wouldn’t steal. | 1 | 2 | 3 | 4 | 5 | 6 |
| 1. Only a coward would ever walk away from a fight. | 1 | 2 | 3 | 4 | 5 | 6 |
| 1. Stores make enough money that’s OK to just take the things you need. | 1 | 2 | 3 | 4 | 5 | 6 |
| 1. A lie doesn’t really matter if you don’t know that person | 1 | 2 | 3 | 4 | 5 | 6 |
| 1. Everybody breaks the law, it’s no big deal. | 1 | 2 | 3 | 4 | 5 | 6 |
| 1. Taking a car doesn’t really hurt anyone if nothing happens to the car and the owner gets it back | 1 | 2 | 3 | 4 | 5 | 6 |
| 1. I can’t help losing my temper a lot. | 1 | 2 | 3 | 4 | 5 | 6 |
| 1. You can’t trust people because they will always lie to you | 1 | 2 | 3 | 4 | 5 | 6 |
| 1. It’s no use trying to stay out of fights. | 1 | 2 | 3 | 4 | 5 | 6 |
| 1. No matter how hard I try, I can’t help getting in trouble. | 1 | 2 | 3 | 4 | 5 | 6 |
| 1. If you don’t push people around, you will always get picked on. | 1 | 2 | 3 | 4 | 5 | 6 |
| 1. People are always trying to hassle me | 1 | 2 | 3 | 4 | 5 | 6 |
| 1. You should hurt people first, before they hurt you | 1 | 2 | 3 | 4 | 5 | 6 |
| 1. You might as well steal. If you don’t take it, somebody else will | 1 | 2 | 3 | 4 | 5 | 6 |
| 1. You might as well steal. People would steal from you if they had the chance. | 1 | 2 | 3 | 4 | 5 | 6 |
| 1. I might as well lie – when I tell the truth, people don’t believe me anyway | 1 | 2 | 3 | 4 | 5 | 6 |
| 1. Everybody steals – you might as well get your share. | 1 | 2 | 3 | 4 | 5 | 6 |

**Time 3**

**PART A: pERSONAL INFORMATION**

QID:______________

Time: T3

| **1.** | Please write the initials of your first and last name followed by your birth month  (e.g. Majid Khan born in July, M.K.07) | | | | |  |  |  | |  |
| --- | --- | --- | --- | --- | --- | --- | --- | --- | --- | --- |
|  | | | | | | | | | | |
| **2.** | **Email / Mobile** | |  | | | | | | | |
|  | | | | | | | | | | |
| **3.** | **Gender** | | Male | 1 | Female | | | | 2 | |
|  | | | | | | | | | | |
| **4.** | | **Marital status** | single | 1 | married | | | | 2 | |
|  |  |  | divorce | 3 | widowed | | | | 4 | |
|  | |  |  |  |  |  |  |  |  |  |
| **5.** | | **Age Group** | Below 25 | 1 | 26 – 35 years | | | | 2 | |
|  |  |  | 36 - 45 years | 3 | 46 – 55 years | | | | 4 | |
|  |  |  | 56 and above | 5 |  | | | |  | |
|  | |  |  |  |  | | | |  | |
| **6.** | | **Educational qualification** | Bachelors | 1 | Masters | | | | 2 | |
|  |  |  | MS/ M.Phil | 3 | Ph.D. | | | | 4 | |
|  |  |  | Post Doctorate | 5 |  | | | |  | |
|  | | | | | | | | | | |
| **7.** | | **Area of specialization** | HR | 1 | Marketing | | | | 2 | |
|  |  |  | Banking & Finance | 3 | IT and Software | | | | 4 | |
|  |  |  | Electric/Civil Engineering | 5 | Medical sciences | | | | 6 | |
|  |  |  | Law and taxation | 7 | Project Management | | | | 8 | |
|  |  |  | Others (please specify) | 9 |  | | | | | |
|  | |  |  |  |  | | | |  | |
| **8.** | | **Type of organization** | Government | 1 | semi government | | | | 2 | |
|  |  |  | private | 3 | MNC | | | | 4 | |
|  |  |  | Other(please specify)_____________________________________ | | | | | | | |
|  | |  |  | | | | | | | |
| **9.** | | **Name of your organization** |  | | | | | | | |
|  | | | | | | | | | | |
| **10.** | | **Your designation/grade**  **(Specify appropriate or equivalent)** | Chairman/GM | 1 | Director | | | | 2 | |
|  |  |  | Dy. Director | 3 | Assistant Director | | | | 4 | |
|  |  |  | Sr Manager/Manager | 5 | Assistant Manager | | | | 6 | |
|  |  |  | Executive | 7 | Officer | | | | 8 | |
|  | |  |  |  |  | | | |  | |
| **11.** | | **Department you are currently working in** | Admin | 1 | HR | | | | 2 | |
|  |  |  | Marketing | 3 | Finance | | | | 4 | |
|  |  |  | IT | 5 | Procurement | | | | 6 | |
|  |  |  | Production | 7 | R & D | | | | 8 | |
|  |  |  | Other(please specify)_____________________________________ | | | | | | | |
|  | |  |  |  |  | | | |  | |
| **12.** | | **Total Working Experience** | Less than 2 Years | 1 | 2 to 5 years | | | | 2 | |
|  |  |  | 6 to 10 years | 3 | More then 10 years | | | | 4 | |
|  | | | | | | | | | | |
| **13.** | | **How long have you been working with your present company** | Less than 2 Years | 1 | 2 to 5 years | | | | 2 | |
|  |  |  | 6 to 10 years | 3 | More then 10 years | | | | 4 | |

| 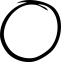  Please circle the appropriate number for your response | Strongly Disagree | Disagree | Slightly Disagree | Slightly Agree | Agree | Strongly Agree |
| --- | --- | --- | --- | --- | --- | --- |
|  | | | | |  | |
| **Does your Co-worker act in a following way?**  **Self serving counterprouductive work behaiovrs by Zahid (2019).** | | | | | | |
| 1. This coworker extended favors to colleagues or higher-ups this coworker to receive some payment or a favor in return | 1 | 2 | 3 | 4 | 5 | 6 |
| 1. tried to hide his/her own errors by masking them covertly | 1 | 2 | 3 | 4 | 5 | 6 |
| 1. This coworker presented ideas of colleagues in a way that portrayed them as his/her ideas, to get credit for himself/herself | 1 | 2 | 3 | 4 | 5 | 6 |
| 1. This coworker searched through documents belonging to his/her coworkers to see if he/she could use the information against them to gain some personal benefit. | 1 | 2 | 3 | 4 | 5 | 6 |
| 1. This coworker tampered with business documents/results to take a benefit or to create a favorable impression of his/her work. | 1 | 2 | 3 | 4 | 5 | 6 |
| 1. This coworker misrepresented or exaggerated his/her work results to get credit | 1 | 2 | 3 | 4 | 5 | 6 |
| 1. This coworker read confidential information or mail addressed to a coworker to see if it can be manipulated for personal gain | 1 | 2 | 3 | 4 | 5 | 6 |
| 1. This coworker used his/her position or resources to oblige colleagues and took undue advantage of the favor. | 1 | 2 | 3 | 4 | 5 | 6 |
